# Reimagining sustainable fisheries

**DOI:** 10.1371/journal.pbio.3001829

**Published:** 2022-10-17

**Authors:** Jennifer Jacquet, Daniel Pauly

**Affiliations:** 1 Department of Environmental Studies, New York University, New York, New York, United States of America; 2 Institute for the Oceans and Fisheries, The University of British Columbia, Vancouver, British Columbia, Canada; Smithsonian Institution, UNITED STATES

## Abstract

Wild animals cannot simultaneously be sustainably exploited and supply growing global markets. This Perspective argues that the current definition of ‘sustainable fisheries’ should be reimagined to minimize exploitation and prioritize small-scale artisanal and subsistence fishing.

## Teaser

The current conception of sustainable fisheries focuses on single “stocks” targeted by industrial fisheries to supply growing global markets, including those for fishmeal. Sustainable fisheries should be reimagined to minimize exploitation and prioritize artisanal and subsistence fishing that feeds people.

Industrial fisheries, which currently account for approximately 75% of global catch [1], began in the 1890s, when the UK deployed the first steel-hulled steam trawlers in its coastal waters. These capital- and energy-intensive behemoths, within 2 decades, decimated costal fish populations around the British Isles and so moved their operations further and further offshore. This was the start of the globalization of industrial fishing, driven by a recurring pattern of fisheries collapses, and compensatory geographic expansion.

One century later, researchers demonstrated that industrial fisheries had a devastating impact on fish populations globally [2–4]. Governments, civil society organizations, university researchers, international bodies, and the private sector responded to rampant overfishing by promoting “sustainable fisheries,” i.e., fisheries operating such that their catch could be maintained indefinitely. However, despite discussions about ecosystem-based management, each of these groups defined (and, to some extent, implemented) the sustainability of fisheries primarily as a management goal to enable a maximal exploitation of single “stocks” of wild aquatic animals, rather than the maintenance of the ecosystems in which these “stocks,” or rather populations, are embedded. The emphasis on the management of single stocks has meant that the concept of sustainable fisheries has been too narrow to achieve commonsense notions of sustainability, given the well-documented propensity of industrial gears, such as trawling, the industrial gear par excellence, to strongly degrade marine ecosystems. The focus on quota setting has come at the cost of broader considerations about delegitimizing destructive fishing practices, restoring ecosystems, addressing overcapacity, eliminating fisheries subsidies, reducing impacts on climate change, and understanding the lives of the animals we exploit and our relationship to them.

Many fisheries labeled as “sustainable” will not be sustained due, e.g., to the modifications they inflict on the ecosystems. Consider the Marine Stewardship Council (MSC), the leading and most visible global fisheries certification scheme, as an example. The MSC has certified the Gulf of Maine lobster fishery, which has recently achieved consistent catches. But the fishery exists in its present state because previous fisheries have collapsed the Gulf of Maine ecosystem, which used to be dominated by Atlantic cod that preyed on lobster. The Gulf of Maine lobster fishery is the result of a highly degraded, extremely vulnerable ecosystem—a “gilded trap” that prevents a bolder vision of ecosystem restoration [5].

The end use of a fishery should also be part of the sustainability conversation. The MSC does not consider end use an important criterion, which is why it certifies numerous fisheries whose catch is reduced to fishmeal. The MSC have certified Norwegian fishing vessels that catch krill in Antarctica that are fed to farmed salmon, which, in turn, are destined for luxury markets [6]. If French companies were killing penguins to fatten the geese used in foie gras, the public would balk at the idea of “sustainably caught penguins” regardless of any outcome suggested by a penguin stock assessment. A large fraction of the industrial fish catch (approximately 20 million tonnes annually between 1950 and 2010) does not feed humans at all but is used as feed for pigs, chickens, and farmed fish, although most of the fish used as animal feed are perfectly edible [7].

The UN Food and Agriculture Organization (FAO), whose primary mission is to eradicate hunger, food insecurity, and malnutrition, is in a strong position to commission a study and create data sets about the end use and who eats the global catch. Broad claims of that industrial fisheries contribute to “food security” often lack empirical evidence (e.g., [8]), while artisanal and subsistence fisheries, which do contribute to the nutrition of rural communities in the Global South, are usually overlooked by national reporting systems (e.g., [9]). An empirical study on where the global catch of aquatic animals actually contributes to food security would be a welcome contribution.

Ending government handouts to industrial fisheries that, e.g., lower the cost of fossil fuel use and vessel construction [10], as well as putting an end to forced labor practices should be, and for some groups, remain, a top priority of the ocean conservation community at the national and international level. Many industrial fisheries, given the overfished state of the marine ecosystems, cannot maintain their operations without these subsidies.

We also need to learn more about exploited aquatic animals beyond how quickly their populations grow. What their lives are like? How do they experience the world? Enterprising graduate students might work to understand tuna cognition and agency as a complement to the vast amount of research into understanding tuna population dynamics.

A reimagining of sustainable fisheries is an exciting prospect. Imagine an ocean in which aquatic animals were protected from industrial fishing. Imagine that strong norms and rules developed against the use of fish and aquatic invertebrates as feed for other animals. Imagine eliminating subsidies, greatly limiting the international trade of these animals, and reserving the right to fish to artisanal fishers supplying local communities and to subsistence fishers providing for their families. Imagine, in other words, that wild fish and invertebrates were considered something more like wild animals and less like traded commodities.

If this vision seems unrealistic or idealistic, consider the whales. The International Whaling Commission (IWC) was formed after World War II to manage the exploitation of whales, and they spent many years designing a management system that would result in sustainable whaling. Yet, whale populations continued to decline. Throughout the 1970s, there was highly visible science, activism, popular media coverage, tightening of national regulations, and international proposals related to anti-whaling, which led to an international moratorium on whaling being first articulated at the 1972 Stockholm Environment Conference [11].

At a special meeting of the IWC Scientific Committee in 1980, a conversation that was largely dominated by stock assessments took an important turn. The meeting focused on “Ethics and Intelligence” and researchers spoke about whale cognition, perception, songs, and our moral duties. Sidney Holt, a population biologist who was influential in our understanding of whale population dynamics [12], spent 2 decades on the IWC scientific committee. Through exposure to other lines of evidence and argument, Holt became convinced that whales had minds and cultures. At the 1982 meeting of the IWC, Holt argued it would be “a great evil to destroy something we don’t understand” [13]. That year, member states voted to adopt a moratorium on whaling, which phased out commercial whaling by 1986 [11].

Whales were once seen as “fish” and managed as a commodity; indeed, the IWC still defines whales as “natural resources.” Yet, currently, this view of whales has few adherents. The large-scale decommodification of whales did not occur by fine-tuning management, but by expanding global consciousness, which resulted in new laws, social norms, and a new relationship. Industrial whaling has, statistically speaking, ceased. Research into just a few species of cetaceans (e.g., dolphins, orcas, humpbacks) was considered sufficient to change policies and perceptions across the 90 species of cetaceans.

To encourage such a shift for the thousands of species of aquatic animals currently exploited for seafood, we know that we will need new kinds of assessments, evidence, and moral arguments beyond stock assessments, spatial distribution, and quota setting. A more expansive view and empirical analysis of sustainable fisheries and fish themselves may help transform our view of wild fish and aquatic invertebrates from commodities to coinhabitants.

## Abbreviations:

FAO: Food and Agriculture Organization
IWC: International Whaling Commission
MSC: Marine Stewardship Council
